# Proteomic similarities in steroid responsiveness in normal and glaucomatous trabecular meshwork cells

**Published:** 2012-07-20

**Authors:** Kathryn E. Bollinger, John S. Crabb, Xianglin Yuan, Tasneem Putliwala, Abbot F. Clark, John W. Crabb

**Affiliations:** 1 Cole Eye Institute, Cleveland Clinic Foundation, Cleveland, OH; 2 Lerner Research Institute, Cleveland Clinic Foundation, Cleveland, OH; 3 Department of Cell Biology and Anatomy, North Texas Eye Research Institute, University of North Texas Health Science Center, Fort Worth, TX; 4 Departments of Ophthalmology and Molecular Medicine, Cleveland Clinic Lerner College of Medicine of Case Western Reserve University, Cleveland, OH

## Abstract

**Purpose:**

Glucocorticoids (GCs) are common anti-inflammatory agents that can cause ocular hypertension and secondary glaucoma as a consequence of impaired aqueous humor outflow through the trabecular meshwork (TM). Mechanisms of GC-signaling are complex and poorly understood. To better understand GC-signaling in the eye, we tested the hypothesis that common mechanisms of steroid responsiveness exist in TM cells from normal and glaucomatous donors.

**Methods:**

Four primary cultures of human TM cells from normal and glaucomatous donors were treated with or without dexamethasone (Dex) for 10 days, then cellular proteins were extracted, identified and quantified by liquid chromatography tandem mass spectrometry (LC MS/MS) iTRAQ (isobaric tags for relative and absolute quantitation) technology.

**Results:**

A total of 718 proteins were quantified. Dex-treatment significantly altered the abundance of 40 proteins in ≥3 cell samples, 37 of which have not previously been associated with GC-signaling in TM cells. Most steroid responsive proteins were changed in all four TM cells analyzed, both normal and glaucomatous. GC-induced proteomic changes support remodeling of the extracellular matrix, disorganization of the cytoskeleton/cell-cell interactions, and mitochondrial dysfunction. Such physiologic consequences appear common to those induced in TM cells by transforming growth factor-β_2_, another putative contributor to ocular hypertension and glaucoma pathology.

**Conclusions:**

The results expand the repertoire of TM proteins involved in GC-signaling, demonstrate common consequences of GC-signaling in normal and glaucomatous TM cells, and reveal similarities in proteomic changes induced by steroids and TGFβ_2_ in normal and glaucomatous TM cells. Finally, the data contributes to a TM quantitative proteomic database.

## Introduction

**Figure 1:**
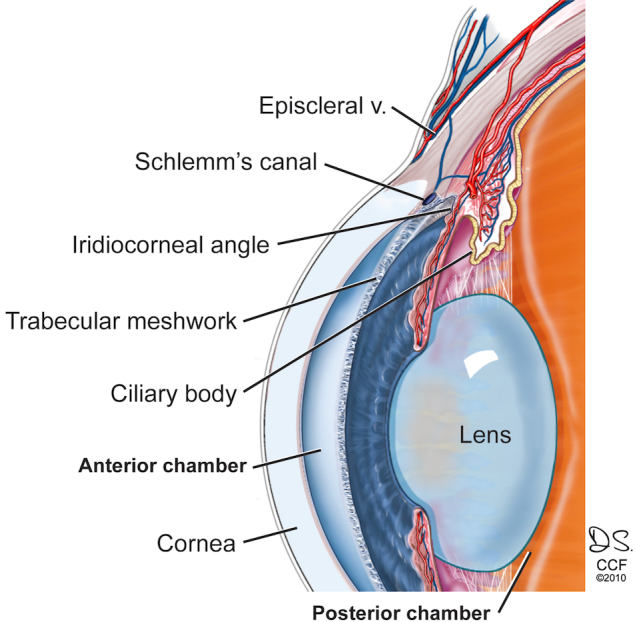
Human trabecular meshwork. Aqueous humor (AH) is actively produced by the ciliary epithelium in the posterior chamber of the eye and circulates through the pupil to the anterior chamber where it drains through the TM into Schlemm’s canal and the episcleral veins. Reproduced with copyright permission from the Cleveland Clinic. Illustration by David Schumick. All rights reserved.

Glucocorticoids (GCs) are potent anti-inflammatory agents used successfully to treat a variety of diseases, but with several potentially serious side effects. In the eye, GC therapy can cause ocular hypertension and secondary open-angle glaucoma [1,2]. About 40% of the general population will develop elevated intraocular pressure (IOP) within 4–6 weeks of topical ocular administration of GCs [3]. Such “steroid responders” are more likely to develop primary open angle glaucoma (POAG) than non-responders. The trabecular meshwork (TM), located in the aqueous humor outflow pathway (Figure 1), regulates IOP through alteration of aqueous humor resistance via contractile properties, phagocytosis, cytoskeletal reorganization, cell adhesion, matrix deposition and ion channel transport [4,5]. The molecular mechanisms causing GC-induced ocular hypertension and impaired TM cell function are not well understood [5,6].

GC-signaling mechanisms appear to be in part tissue-specific [7] and highly complex [8], with hundreds of gene expression changes induced in TM cell cultures by dexamethasone (Dex) [9-13]. Many of the GC-induced changes in the TM are similar to those seen in POAG [3]. GC-induced ocular hypertension occurs in both normal and glaucoma patients, although a higher percentage of glaucoma patients are steroid responsive. The GC-induced changes to the TM, the resulting elevation in IOP, and the clinical phenotype appear to be similar in these two groups. The goal of this study was to test the hypothesis that common mechanisms of steroid responsiveness exist in TM cells from normal and glaucomatous tissues. Primary TM cells cultured from carefully dissected TM tissues were used for global quantitative proteomic analysis of steroid responsiveness using liquid chromatography tandem mass spectrometry (LC MS/MS) isobaric tags for relative and absolute quantitation (iTRAQ) technology. Although cultured TM cells grow very slowly and have a limited proliferative capacity, we were able to examine GC-induced changes in protein expression in four different primary TM cell cultures, more than any previous study of GC effects on gene or protein expression in the TM. The results identfy a significant number of proteins not previously known to be steroid responsive in TM cells and show most of the GC-altered proteins were changed in all TM cell strains analyzed, both normal and glaucomatous.

## Methods

### TM cell cultures

All human specimens were post-mortem tissues collected with adherence to the principles expressed in the Declaration of Helsinki. Post-mortem human eyes were obtained from the Lions Eye Institute for Tissue and Research in Tampa, FL, which is accredited by the Eye Bank Association of America. TM cells were isolated from TM tissue explants derived from both open angle glaucoma and nonglaucomatous control donors. The glaucoma status was indicated from donor medical histories. The average death to preservation time was 7.7±3.3 h. Eyes were stored at 4 °C until the TM was dissected, generally within 24–36 h. Primary cultures were established and TM cell morphology and purity were characterized as previously described [14,15]. Human TM cells were grown in Dulbecco’s modified Eagle’s medium (HyClone, Logan UT) supplemented with 10% fetal bovine serum (GIBCO, Grand Island, NY), 1% penicillin-streptomycin (HyClone) and 1% L-glutamine (Thermo-Scientific Hyclone, Logan, UT) to confluency in T-25 flasks or in 6-well plates. Primary cultures of human TM cells from 2 POAG and 2 non-glaucomatous donors were treated with or without Dex (100 nM) for 10 days, yielding 4 Dex-treated and 4 untreated TM cell cultures (Table 1).

**Table 1:** Trabecular meshwork cell samples.

**Experiment**	**Cell culture**	**Donor age and gender**	**Cell passage**	**Protein analyzed µg**
1	NTM416-07	78 / M	P4	75
2	NTM496-05	82 / F	P3	75
3	GTM304-04	75 / F	P3	75
4	GTM477-02	85 / F	P4	75

Each of the above primary TM cell cultures was treated with or without Dex then analyzed by LC MS/MS iTRAQ technology. NTM, normal trabecular meshwork; GTM, glaucomatous trabecular meshwork; M, male; F, female; P, cell passage.

### iTRAQ labeling, SCX chromatography, protein identification, quantitation, and bioinformatics

Detailed methods for sample preparation, iTRAQ labeling, strong cation exchange chromatorgraphy (SCX), protein identification and quantitation and bioinformatics have been described elsewhere [16-18]. Briefly, for proteomic analyses, proteins were extracted from TM cells [18], quantified by amino acid analysis [19], reduced, alkylated, and digested with trypsin [18]. Tryptic peptides from Dex-treated TM cells were labeled with iTRAQ tag 117 and mixed with an equal amount of tryptic peptides from the corresponding untreated cell sample labeled with iTRAQ tag 115. Each peptide mixture was fractionated by strong cation exchange (SCX) chromatography and fractions collected for LC MS/MS. LC MS/MS was performed with a QTOF2 mass spectrometer equipped with a Cap LC system (Waters Corporation, Milford, MA). Protein identification used MASSLYNX 4.1 software (Waters), the Mascot search engine (Matrix Science, Boston, MA), and the SwissProtein human sequence database (version 56.0, ~20,000 total sequences). Two unique peptides per protein and Mascot peptide ion scores ≥25 were required for all protein identification and quantitation. To estimate false discovery rates, all peptide MS/MS spectra were searched (Matrix Science, version 2.2) against a randomized decoy database constructed from the above SwissProtein database with a script provided by Matrix Science [16-18]. Protein quantitation from iTRAQ labeling required ion intensities ≥10 for all iTRAQ tags and was achieved with code written in the statistical program R. To average results over multiple samples, protein ratios were normalized to the protein median per sample, then average protein ratios (unadjusted), standard errors of the mean (SEM) and p-values (two sided t-Test for the null hypothesis that the protein mean=0 in log space) were calculated. Average protein ratios were adjusted to give greater weight to data with lower SEM values then adjusted SEMs and p-values were determined from the adjusted means of the multiple measurements [16-18]. Bioinformatic analyses were performed with The **P**rotein **AN**alysis **TH**rough **E**volutionary **R**elationships (PANTHER) Classification System, Ingenuity Pathways Analysis 8.5 (Ingenuity Systems, Redwood City, CA), and the SwissProtein database.

### Western Analyses

Immunoblots were performed as previously described [18]. Briefly, TM cell cultures were washed with PBS twice, and proteins were extracted with Mammalian Protein Extraction Buffer (ThermoScientific) containing 1% protease inhibitor cocktail (Thermo Fischer Scientific, Rockford, IL). Protein concentrations in TM lysates were determined by the BioRad Dc Protein Assay (Bio-Rad Laboratories, Hercules, CA). SDS-PAGE was performed on 10% acrylamide gels with 30 µg protein applied per lane, and proteins were electrophoretically transferred to polyvinylidene fluoride membranes (EMD Millipore, Billerica, MA). Membranes were blocked with 5% non-fat dry milk in TBST buffer then incubated overnight at 4 °C with primary antibody. The membranes were washed with TBST and probed with horseradish peroxidase-conjugated secondary antibody in 3% non-fat milk in TBST for 1 h at room temperature. Primary antibodies included mouse monoclonal anti-activated leukocyte cell adhesion molecule antigen (CD166; ALCAM; Novacastra/NCL-CD166, diluted 1:40; Abcam, Cambridge, MA) and rabbit polyclonal anti-Tplastin (Plastin-3; #ab45769, diluted 1:1,000; Abcam). Rabbit monoclonal anti-glyceraldehyde-3-phosphate dehydrogenase was used as a loading control (#14C10, diluted 1:1,000; Cell Signaling/Millipore, Billerica, MA). Secondary antibodies included horseradish peroxidase-conjugated secondary antibody (goat anti-mouse IgG; #sc2005, diluted 1:10,000; Santa Cruz, Santa Cruz, CA) or goat anti-rabbit IgG (#7074 diluted 1:10,000; Cell Signaling). Proteins were detected using enhanced chemiluminescence and a Fluor ChemTM 8900 imager (Alpha Innotech/ProteinSimple, Santa Clara, CA). Immunoreactivity was quantified using Image J software (NIH), and statistical analyses were performed using the two-sided *t*-test.

## Results

**Figure 2:**
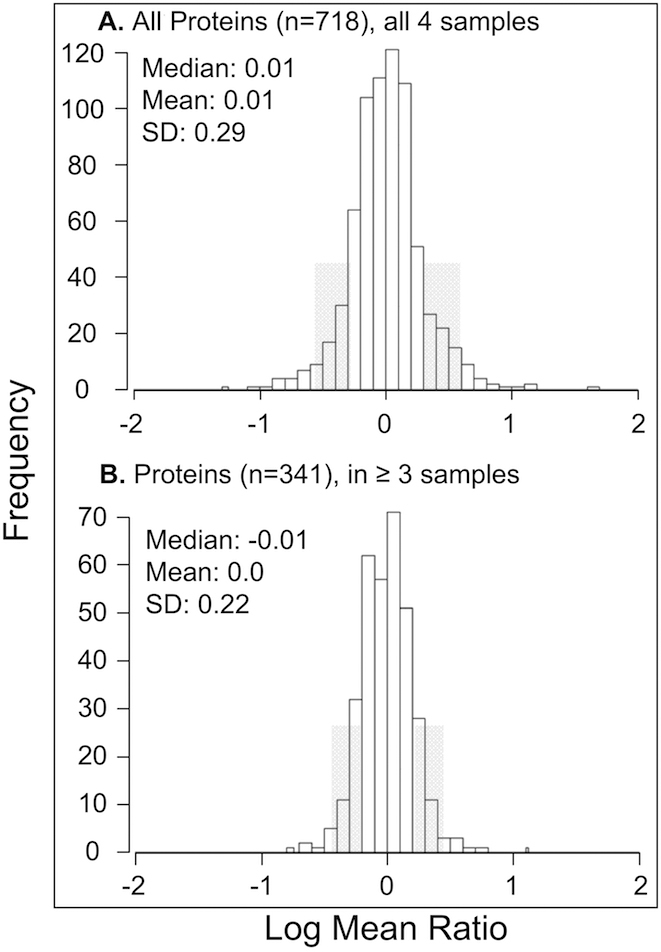
Distribution of protein ratios. The log_2_ mean distribution of protein ratios (Dex-treated TM/untreated TM) are shown for all 718 quantified proteins and 341 proteins quantified in ≥3 cell samples. Median, mean and SD values are indicated; protein ratios between 1 and 2 SD from the mean are shaded. The distribution of protein ratios is near-to-normal.

A total of 718 proteins were quantified by LC MS/MS iTRAQ technology from four primary cultures of human TM cells treated or not treated with Dex. TM cell sample properties are defined in Table 1. Quantitative proteomics results from each sample are presented in Appendix 1, Appendix 2, Appendix 3, and Appendix 4, including protein ratios, SDs, p-values, number of unique peptides quantified, percent sequence coverage for each protein, and peptide false discovery rates. The data from all four TM samples were of comparable quality and appropriate for averaging based on consistently low peptide false discovery (average rates, 1.6% identity, 2.7% homology) and similar distributions of protein ratios. The average relative abundance of all 718 quantified proteins is itemized in Appendix 5. The distributions of log_2_ mean protein ratios for all proteins, and those quantified in ≥3 TM samples (n=341), are shown in Figure 2. The normal distributions (Figure 2) support approximately equal numbers of proteins increased or decreased by Dex-treatment. Criteria for determining whether a protein was elevated or decreased by Dex-treatment included the average adjusted protein ratio and p-value with only proteins quantified in ≥3 cell samples used for comparative purposes. Proteins exhibiting average protein ratios (adjusted by SEM) above or below the mean by at least 1 SD (Figure 2) and p-values ≤0.055 were considered of higher or lower abundance.

### The majority of the determined proteome was unchanged by Dex-treatment

About 94% of all 718 proteins and ~88% of the 341 proteins quantified in three or more samples appear to be present in similar amounts in Dex-treated and untreated cells (Figure 2 and Appendix 5). Accordingly, the determined proteome largely reflects that of untreated TM cells. The quantified proteins include ~6% secreted, ~48% cytoplasmic, ~22% membrane and membrane-associated, ~15% nuclear, and ~11% mitochondrial proteins. These proportions are very similar to that obtained in quantitative proteomic analysis of the same TM cells treated with transforming growth factor beta 2 (TGFβ_2_) [18]. In the current study, Dex-treatment decreased the abundance of more secreted proteins (n=3) than it increased (n=0) and elevated the abundance of more mitochondrial proteins (n=6) than it decreased (n=2).

### TM proteins increased by Dex

Twenty proteins were elevated ≥1 SD above the mean ratio (p<0.05) in ≥3 samples by Dex-treatment, including both normal and glaucomatous TM cells (Table 2). Six proteins were elevated ≥2 SD by Dex treatment. Elevated proteins were associated with the cytoskeleton, ECM, mitochondrial metabolism and carbohydrate metabolism. The majority of these proteins have not previously been associated with GC-signaling in TM cells.

**Table 2:** Proteins elevated in dexamethasone-treated TM cells.

**SwissProt accession**	**Protein**	**Subcellular source**	**Sample frequency total=4**	**Mean protein ratio**	**SEM**	**p-value**
**Cytoskeletal/cell-cell/cell-matrix Interactions**						
O94875	Sorbin and SH3 domain-containing protein 2	B	4	3.06*	0.10	0.001
Q0ZGT2	Nexilin	B	4	2.03*	0.17	0.026
Q13740	CD166 antigen	C	4	1.99*	0.12	0.011
O75369	Filamin-B	B	4	1.64*	0.11	0.020
Q15942	Zyxin	BD	4	1.38	0.04	0.005
Q13418	Integrin-linked protein kinase	C	4	1.36	0.09	0.037
Q8WX93	Palladin	B	4	1.31	0.08	0.044
P35613	Basigin	C	4	1.29	0.04	0.007
Q6NZI2	Polymerase I and transcript release factor	BC	4	1.26	0.05	0.020
**Carbohydrate Metabolism**						
P37837	Transaldolase	B	4	1.43	0.08	0.019
P52209	6-phosphogluconate dehydrogenase, decarboxylating	B	4	1.35	0.07	0.019
P11413	Glucose-6-phosphate 1-dehydrogenase	B	3	1.30	0.03	0.015
P60174	Triosephosphate isomerase	B	4	1.26	0.06	0.031
**Mitochondrial Metabolism**						
Q9Y6N5	Sulfide:quinone oxidoreductase	E	4	1.81*	0.06	0.002
Q9UIJ7	GTP:AMP phosphotransferase	E	4	1.56*	0.07	0.007
P42765	3-ketoacyl-CoA thiolase	E	4	1.39	0.06	0.011
Q13510	Acid ceramidase	B	3	1.35	0.05	0.031
P30049	ATP synthase subunit delta	CE	4	1.34	0.07	0.023
P61604	10 kDa heat shock protein	E	4	1.33	0.08	0.042
P36957	Dihydrolipoyllysine-residue succinyltransferase component of 2-oxoglutarate dehydrogenase complex	E	3	1.25	0.03	0.014

Adjusted mean protein ratios (DEX-treated/Control), standard error of the mean (SEM), p-values and subcellular source are shown for abundant proteins (from Appendix 5). Abundant proteins exhibited mean ratios at least 1SD above the mean and p-values <0.05 (see Figure 2). All proteins were quantified in ≥3 TM cell samples (median ratio=0.99, mean=1.00, SD=0.22, n=341). Asterisks denote ratios at least 2 SD above the mean. Protein subcellular source from the Swiss Protein database: A, secreted; B, cytoplasmic; C, membrane; D, nuclear; E, mitochondrial.

### TM proteins decreased by Dex

Twenty proteins were significantly decreased ≥1 SD below the mean ratio (p≤0.55) in ≥3 samples by Dex-treatment, including 4 reduced ≥2 SD (Table 3), and all were changed in both normal and glaucomatous TM cells. Decreased proteins were associated with stress response, cellular defenses, protein processing, the cytoskeleton, the extracellular Matrix (ECM), and mitochondrial metabolism. The majority of the Dex-decreased proteins have not previously been considered steroid responsive in TM cells.

**Table 3:** Proteins decreased in dexamethasone-treated TM cells.

**SwissProt accession**	**Protein**	**Subcellular source**	**Sample frequency total=4**	**Mean protein ratio**	**SEM**	**p-value**
**Stress Response, Cellular Defense and Protein Processing**						
P17931	Galectin-3	ABD	3	0.80	0.05	0.054
P25786	Proteasome subunit alpha type-1	BD	3	0.79	0.06	0.051
Q96AY3	FK506-binding protein 10**	C	4	0.78	0.04	0.009
Q14697	Neutral alpha-glucosidase AB	C	3	0.77	0.06	0.042
P06703	Protein S100-A6**	BCD	4	0.75	0.03	0.003
P27797	Calreticulin**	ABC	4	0.73	0.09	0.039
P05388	60S acidic ribosomal protein P0	B	3	0.73	0.02	0.003
O95302	FK506-binding protein 9**	C	4	0.71	0.06	0.010
P23284	Peptidyl-prolyl cis-trans isomerase B	C	4	0.69	0.11	0.045
P30040	Endoplasmic reticulum protein ERp29	C	4	0.66	0.05	0.004
P50454	Serpin H1**	C	4	0.52*	0.07	0.003
**Cytoskeletal and ECM Interactions**						
P13797	Plastin-3**	B	4	0.73	0.10	0.048
O15460	Prolyl 4-hydroxylase subunit alpha-2	C	4	0.75	0.09	0.053
Q01995	Transgelin**	B	4	0.78	0.07	0.042
P13674	Prolyl 4-hydroxylase subunit alpha-1	C	4	0.78	0.08	0.055
P48681	Nestin	B	3	0.62*	0.04	0.007
P02452	Collagen alpha-1(I) chain	A	3	0.48*	0.15	0.038
**Mitochondrial Metabolism**						
P30153	Serine/threonine-protein phosphatase 2A 65 kDa regulatory subunit A alpha isoform	D	3	0.78	0.01	0.004
P22307	Non-specific lipid-transfer protein	BE	4	0.73	0.10	0.049
O75964	ATP synthase subunit gamma	CE	3	0.61*	0.12	0.054

Adjusted mean protein ratios (DEX-treated/Control), standard error of the mean (SEM), p-values and subcellular source are shown for less abundant proteins (from Appendix 5). Less abundant proteins exhibited mean ratios at least 1SD below the mean and p-values ≤0.055 (see Figure 2). All proteins were quantified in ≥3 TM cell samples (median ratio=0.99, mean=1.00, SD=0.21, n=341). Asterisks (*) denote ratios at least 2 SD below the mean. Double asterisks (**) denote CA^++ ^binding proteins. Protein subcellular source from the Swiss Protein database: A, secreted; B, cytoplasmic; C, membrane; D, nuclear; E, mitochondrial.

### Independent evidence supporting the iTRAQ protein quantitation

**Figure 3:**
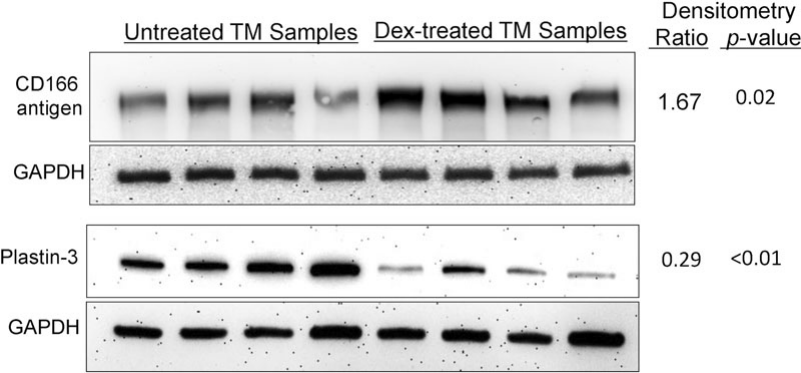
Western analyses. Western blot analysis was used to evaluate the relative amounts of proteins in Dex–treated and untreated TM cells (n=4 samples each). Immunoblot intensities were normalized to GAPDH; average densitometry ratios (Dex–treated/untreated) and p values (two-sided *t*-test) for CD166 antigen (ALCAM) and Plastin-3 support the quantitative data in Table 2 and Table 3.

Western blot analysis (Figure 3) demonstrated Dex-increased amounts of ALCAM/CD166 antigen (p=0.02) and Dex-decreased amounts of plastin-3 (p<0.01), corroborating the iTRAQ protein quantitation. These proteins were selected only because of their presence in Table 2 and Table 3 and the availability of antibodies useful for western analysis. Dex-decreased collagen α1(1) is supported by a previous proteomic study [20] that reported Dex-down-regulation of collagen α1(1) in rat TM (Table 3). Dex-increased amounts of sorbin and SH3 domain containing protein 2 [12] and filamin B [12] are indirectly supported by gene expression studies that reported Dex-induced upregulation of these genes in TM cells.

## Discussion

**Figure 4:**
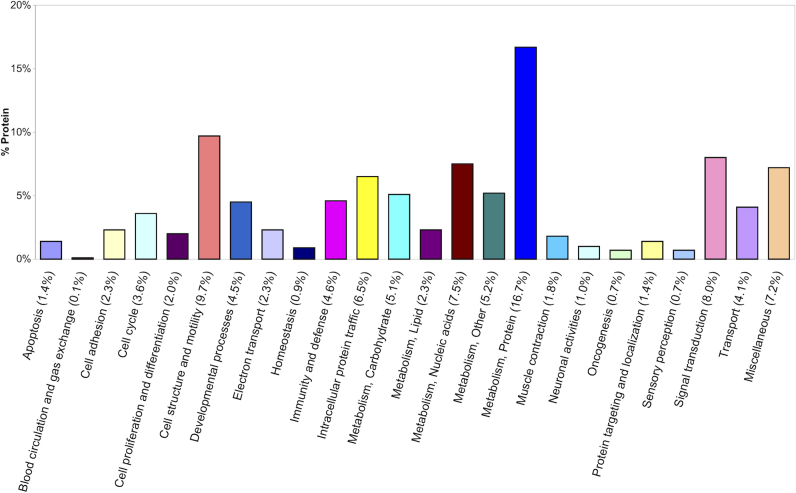
Biologic functions of TM proteins. Functional analysis of the 718 proteins quantified in human TM cells was performed using the PANTHER Classification System.

To better understand GC-signaling in TM cells, we quantified proteins in four primary cultures of human TM cells with and without Dex-treatment using LC MS/MS iTRAQ technology. The study goal was to test the hypothesis that common mechanisms of steroid responsiveness exist in TM cells from normal and glaucomatous tissues. Differences in the levels of glucocorticoid receptor isoforms between normal and glaucomatous cells have been suggested to contribute to steroid responsiveness in TM cells [21,22]. However, similarities in clinical phenotypes of GC-induced ocular hypertension in normal and glaucoma patients suggest common mechanisms may also contribute to steroid responsiveness. Global proteomic analysis of TM cells from both donor populations has provided evidence for common molecular consequences of GC–signaling in non-glaucomatous and glaucomatous TM cells. Notably, ~72% of Dex-altered proteins in this study were significantly changed in all four TM cell strains analyzed. The majority of the 718 quantified proteins were present in similar amounts in Dex-treated and untreated cells; therefore, the overall determined proteome reflects that of untreated TM cells. Dex-treatment significantly altered the abundance of 40 proteins; immunoblots independently corroborated two of these changes and literature reports support three other observed proteomic changes induced by Dex. Thirty-seven of the 40 altered proteins have not been previously recognized as steroid responsive in TM cells. Major biologic processes associated with the determined proteome (Figure 4) include cellular metabolism (37% of the 718 proteins), signal transduction (8%), cell structure/motility (10%), intracellular traffic (7%), and immunity and defense (5%). Ingenuity Pathway analysis of the proteome implicates protein synthesis, cell death, and post-translational modification as the highest scoring networks.

### Upregulated GC-responses in normal and glaucomatous TM cells

Twenty TM proteins were significantly increased in abundance following Dex-treatment (Table 2), supporting steroid-induced, upregulated expression of these polypeptides. Pathway analysis of these proteins highlights cellular movement, cell death, and cell morphology as one of the two highest scoring networks. Several cytoskeletal proteins were elevated, including filamin-B, which connects the cell membrane with the actin cytoskeleton and promotes actin filament branching. These findings are consistent with other studies showing that Dex stimulates an increase in cross-linked actin networks (CLANs) in cultured human TM cells [14]. Such morphological networks are also observed in vivo in both normal and glaucomatous TM [23]. Sorbin and SH3 domain-containing protein 2, elevated about threefold, interacts with a large number of proteins, linking signaling complexes with the cytoskeleton. Increased levels of focal adhesion proteins palladin, zyxin, integrin-linked protein kinase (ILK) and the adherens junction protein nexilin, are consistent with GC-stimulated formation of adherens junctions and tight junctions, previously observed in TM cells [24] and in mammary tumor cells [25]. Notably, ILK functions in the regulation of cell-cell and cell-matrix interactions and appears to have roles in both integrin and growth factor-signaling. Two other elevated proteins potentially impacting cell adhesion and cell motility were CD166 antigen (ALCAM) and basigin. Both are plasma membrane proteins that promote growth of cellular processes and activate leuckocytes. CD166 antigen also functions in cellular interactions in the nervous system [26] and basigin (also known as tumor cell-derived collagenase stimulatory factor) stimulates matrix metalloproteinase production and tumor progression. Finally polymerase 1 and transcript release factor was elevated ~26% by Dex treatment. This protein is essential for caveolae formation in mammals, and caveolae function in cell proliferation, cargo-specific endocytosis, and mechanosensation [27]. Overall, these 9 elevated proteins support the hypothesis that GC-treatment can alter TM cytoskeletal and cell-cell interactions that contribute to AH outflow resistance and elevated IOP [3].

The other high scoring network from pathway analysis highlights cell cycle, carbohydrate, and nucleic acid metabolism. Consistent with GC regulation of gluconeogenesis [28], four enzymes in the pentose-phosphate pathway were upregulated ~30%–40%, namely transaldolase, 6-phosphogluconate dehydrogenase, glucose-6-phosphate dehydrogenase, and triosephosphate isomerase. The consequences of disrupting the pentose shunt in the TM remain to be determined, but genetic defects in these 4 enzymes can cause severe disorders elsewhere in the body, including progressive neuromuscular dysfunction.

Six mitochondrial proteins and a 7th protein that could significantly alter mitochondrial functions were elevated by Dex-treatment. These mitochondrial proteins serve a variety of roles in energy production (ATP synthase δ), signal transduction (GTP:AMP phosphotransferase), oxidation reduction (sulfide:quinone oxidoreductase), chaperone activity (10 kDa heat shock protein), lipid metabolism (3-ketoacyl-CoA thiolase), and carbohydrate metabolism (component of 2-oxoglutarate dehydrogenase complex). These changes could disrupt TM homeostasis by altering oxidative phosphorylation, regulation of apoptosis by reactive oxygen species, and maintenance of intracellular calcium levels [29,30]. Acid ceramidase, the 7th elevated protein, is a cytosolic protein that functions in the sphingosine-ceramide salvage pathway in sphingolipid turnover [31]. Ceramides produced in the salvage pathway in human breast cancer cells accumulate in mitochondria, promote the relocalization of mitochondria around the nuclear envelope and have been linked to apoptosis and mediators of mitochondrial functions [32]. Impaired mitochondria in TM cells has recently been implicated in POAG [18,33,34]. The present results provide clear evidence that GC-signaling can modify mitochondrial protein expression in both normal and glaucomatous TM cells. Dysfunctional TM mitochondria could promote oxidative damage [18,35] and cellular senescence in the aqueous humor outflow pathway [36].

### Down-regulated GC-responses in normal and glaucomatous TM cells

Twenty TM proteins were significantly reduced by Dex-treatment (Table 3), suggesting GC-induced down-regulation, and/or degradation. Pathway analysis of these 20 proteins highlight gene expression, free radical scavenging and molecular transport as the highest scoring networks. TM cells from POAG donors reportedly contain higher levels of reactive oxygen species and Ca^2+^ in the cytosol and the mitochondria [33,34]. Interestingly, ~35% (n=7) of the TM proteins reduced by Dex-treatment were calcium-binding proteins (denoted by ** in Table 3), supporting calcium dysregulation in the TM as possibly contributing to elevated IOP [33]. About 50% of the decreased proteins can be associated with cellular defense, stress response, and processing events, consistent with an anti-inflammatory response. Several of these exhibit protein-folding and chaperone functions, including FK506-binding proteins 9 and 10, calreticulin, peptidyl-prolyl cis-trans isomerase B, endoplasmic reticulum resident protein 29, and Serpin H1. Noteworthy, serpin H1 is a stress-induced, collagen-binding chaperone, and calreticulin functions in the GC receptor signaling pathway. Two damage-associated molecular pattern proteins (DAMPs) were also decreased by Dex, namely advanced glycation endproduct receptor 3 (galectin-3) and protein S100-A6. DAMPs are endogenous proteins released by damaged cells that are capable of binding and activating an inflammatory response through pattern recognition receptors such as the complement system, toll-like receptors, and receptor for advanced glycation endproducts (RAGE). Also decreased was proteasome subunit α type-1, important in the processing of MHC peptides, regulation of the cell cycle, and ubiquitin-dependent degradation processes. Others included the 60S acidic ribosomal protein P0 involved in translational elongation, and neutral α-glucosidase AB which functions in conjunction with calreticulin in the release of mono-glycosylated glycoproteins.

Six proteins impacting the cytoskeleton and/or ECM were decreased by Dex-treatment. Among this group were actin-binding proteins transgelin and plastin-3, as well as nestin, involved in intermediate filament assembly/disassembly and cellular remodeling. Altered levels of these proteins could facilitate GC-induced CLAN formation [14]. ECM protein collagen α1(1) was the most significantly reduced, down ~50%. Two enzymes that catalyze proline hydroxylation in collagen were also decreased, namely prolyl 4-hydroxylase α1 and α2. Collagen α1(1) fibrils are a major component of collagen beams in TM in vivo and at physiologic conditions require hydroxyproline to stabilize the triple-helical structure. Altered interactions between collagen fibrils and other ECM components have been reported to trigger collagen degradation and a loss of tissue-specific morphology [37,38]. Whether the reduced collagen α1(1) level we observed in TM cells is due to decreased expression or to degradation is not clear, but perturbed collagen levels in vivo would render the ECM more susceptible to collapse and debris deposition. Collectively, the observed proteomic changes could disrupt the TM cytoskeleton and associated ECM in both normal and glaucomatous TM.

Dex-treatment also decreased the abundance of proteins impacting mitochondrial metabolism (Table 3). Among these were mitochondrial proteins ATP synthase γ, important in energy production and non-specific lipid-transfer protein, functioning in lipid transport across membranes and possibly serving in steroid biosynthesis. Also decreased was serine/threonine-protein phosphatase 2A isoform PR65-α, which appears to be a regulator in cell adhesion, apoptosis and ceramide-associated processes. As noted, ceramides have been linked to apoptosis and may accumulate in the mitochondria [32]. These results are consistent with GC-inducable mitochondrial dysfunction in both normal and glaucomatous TM cells.

### Comparison with previous studies of GC-signaling in TM cells

This is the most extensive quantitative proteomic analysis of GC-signaling in TM cells. A previous 2D-gel proteomic study of one immortalized TM cell line identified 163 proteins and reported Dex-down regulation of Rho GDP dissociation inhibitor (RhoGDI) [39]. In the present study, four different primary TM cell cultures were analyzed and RhoGDI was not changed by Dex treatment (Appendix 5). Only one other proteomic study of steroid signaling in the anterior segment is currently published. Proteomic changes induced by in vivo topical application of Dex to three rat eyes was investigated using peptide mass fingerprinting methods and 2D fluorescence difference gel electrophoresis [20]. Four Dex-altered TM proteins were reported, including upregulation of αA-crystallin and βA3-crystallin and down-regulation of collagens α1(1) and α2(1) [20]. We found Dex-decreased amounts of collagen α1(1), detected collagen α2(1) in 2 samples, but did not detect αA- and βA3-crystallins, although several other chaperones were decreased by Dex-treatment (Table 2). The earlier report suggested that Dex may induce impaired collagen processing [20] because only COOH-terminal propeptides were detected. This may occur but our study quantified seven internal peptides from throughout the collagen α1(1) sequence, supporting Dex-induced decreased expression of collagen α1(1).

Hundreds of Dex-induced transcript changes in human TM cells have been described in five gene-profiling studies [9-13]; however, only two genes were reported differentially expressed in all five studies, namely genes encoding myocilin and insulin-like growth factor binding protein 2 (both upregulated). We detected myocilin at elevated levels in two of the four TM cell samples (Appendix 1 and Appendix 3) but did not detect insulin-like growth factor binding protein 2. While myocilin is a glaucoma gene, it is not always elevated at the protein level in TM tissues in open angle glaucoma [20]. Myocilin is a secreted glycoprotein, which may account in part for variable TM levels. In our study, myocilin may have also been in the culture medium, which was not analyzed. Among transcripts reported differentially expressed in the TM by Dex [9-13], we found 27 corresponding gene products, from which reliable quantification was obtained on 11 proteins (ie, proteins found in ≥3 samples). Our proteomic data support upregulation of sorbin and SH3 domain containing protein 2 [12] and filamin B [12] but do not support upregulation of transgelin [11,12] nor the reported down-regulation of 3-ketoacyl-CoA thiolase β [9]. Seven other transcripts reported to be differentially expressed exhibited no significant change at the protein level in this study, namely lactate dehydrogenase A [11], clusterin [10], fibroblast muscle tropomyosin [9], skeletal β-tropomyosin [9], fibulin-1C [9], phosphatidylethanolamine binding protein [9], and thrombospondin [13]. The observed differences between proteomic and genomic data seem reasonable as only ~20% correlation generally exists between mRNA and corresponding protein levels in mammals [40,41]. Similar to our findings from quantitative proteomic analysis of TGFβ_2_-signaling in TM cells [18], very few proteases or protease inhibitors were detected in this study (Appendix 5), perhaps because they were below detection limits, and none were found significantly altered by Dex-treatment.

### Additional insight to steroid responsiveness in TM cells

To further probe molecular mechanisms of steroid responsiveness, we compared Dex-induced proteomic changes with previously identified protein changes in TM cells induced by TGFβ_2_ [18]. TGFβ_2_ is elevated in the anterior segment of glaucoma patients, and while the mechanism(s) responsible for elevated expression is unknown, substantial evidence implicates TGFβ_2_ as a contributing factor in ocular hypertension [3,18]. Our TGFβ_2_ investigation [18] used the same normal and glaucomatous TM cell strains and quantitative proteomic methods as used in the present study. For comparison of Dex- and TGFβ_2_-induced proteomic changes, proteins quantified in ≥3 TM cell samples in both studies were sought that exhibited: (i) ratios at least 1 SD above or below the means in both data sets, and (ii) a p-value ≤0.055 in at least one of the treatments (indicating at least one of the treatments significantly changed the protein ratio). Twenty proteins meeting the above criteria were identified (Table 4), including four apparently altered by both TGFβ_2_ and Dex (palladin, prolyl 4-hydroxylase α1 and α2, and FK506-binding protein 10). While additional studies with larger samples sizes are warranted to validate proteins altered by both Dex and TGFβ_2_, these two modifiers appear to impact several common physiologic processes in both normal and glaucomatous TM cells. These processes include cytoskeletal/cell-cell interactions, cell-matrix/ECM remodeling, and mitochondrial metabolism [18]. Such common physiologic consequences suggest a molecular basis for the increased risk of ocular hypertension in steroid responders.

**Table 4:** Proteomic changes induced in TM cells by dexamethasone and TGFβ_2_.

** **	** **	**Dex**		**TGFβ_2_**	
**Swiss prot accession**	**Protein**	**Ratio**	**p value**	**Ratio**	**p value**
**Cytoskeletal and Cell-Cell Interactions**					
Q8WX93	Palladin	↑ 1.3	0.044	↑ 1.7	0.003
Q9HBL0	Tensin-1	↑ 1.7	0.119	↑ 1.7	0.017
P37802	Transgelin-2	↑ 1.3	0.127	↑ 1.6	0.012
Q0ZGT2	Nexilin	↑ 2.0	0.026	↑ 1.4	0.152
Q01995	Transgelin	↓ 0.8	0.042	↑ 1.7	0.151
P13797	Plastin-3	↓ 0.7	0.048	↑ 1.5	0.093
**Cell-Matrix and ECM Remodeling**					
O15460	Prolyl 4-hydroxylase subunit alpha-2	↓ 0.7	0.053	↑ 2.1	0.042
P13674	Prolyl 4-hydroxylase subunit alpha-1	↓ 0.8	0.055	↑ 1.5	0.057
P07996	Thrombospondin-1	↓ 0.7	0.147	↑ 2.4	0.030
P02452	Collagen alpha-1(I) chain	↓ 0.5	0.038	↑ 1.7	0.263
**Mitochondrial Metabolism**					
P04179	Superoxide dismutase [Mn]	↓ 0.8	0.272	↓ 0.5	0.002
Q9UIJ7	GTP:AMP phosphotransferase mitochondrial	↑ 1.6	0.007	↓ 0.7	0.168
P42765	3-ketoacyl-CoA thiolase	↑ 1.4	0.011	↓ 0.7	0.200
Q9Y6N5	Sulfide:quinone oxidoreductase	↑ 1.8	0.002	↓ 0.7	0.067
P00367	Glutamate dehydrogenase 1	↑ 1.3	0.061	↓ 0.6	0.012
O94925	Glutaminase kidney isoform	↓ 0.8	0.206	↑ 1.4	0.037
**Stess Response and Protein Folding**					
Q96AY3	FK506-binding protein 10	↓ 0.8	0.009	↑ 1.5	0.005
Q15084	Protein disulfide-isomerase A6	↓ 0.8	0.092	↑ 1.4	0.041
**Regulatory Processes**					
P50479	PDZ and LIM domain protein 4	↑ 1.3	0.290	↑ 1.7	0.036
Q99536	Synaptic vesicle membrane protein VAT-1 homolog	↓ 0.8	0.561	↓ 0.7	0.004

Proteins are shown that were quanitified in both this study and a study of TGFβ_2_-induced proteomic changes in TM cells [18]. All proteins were quantified in ≥3 TM samples, exhibited ratios at least 1 SD above or below the means, and p-values ≤0.055 in at least one of the treatments (indicating at least one ratio significantly different than 1.0). Arrows reflect elevated or decreased expression.

### Conclusions

Based on our analysIs of a small number of TM cell samples, the results expand the repertoire of proteins participating in GC-signaling and support common steroid response mechanisms in both normal and glaucomatous TM cells. While we analyzed more cell samples than in previous studies of GC effects on the TM transcriptome or proteome, the sample size is limiting and further investigations are warranted. The observed proteomic changes implicate as consequences of steroid-treatment remodeling of the extracellular matrix, disorganization of the cytoskeleton, disruption of cell-cell interactions, and mitochondrial dysfunction in the TM. These same physiologic processes appear impacted by TGFβ_2_-signaling in both normal and glaucomatous TM cells. Finally, the results contribute to a quantitative database of TM proteins.

## Appendix 1. Relative protein abundance: Trabecular meshwork sample NTM416–07.

To access the data, click or select the words “Appendix 1.” This will initiate the download of a compressed (pdf) archive that contains the file.

## Appendix 2. Relative protein abundance: Trabecular meshwork sample NTM496–05.

To access the data, click or select the words “Appendix 2.” This will initiate the download of a compressed (pdf) archive that contains the file.

## Appendix 3. Relative protein abundance: Trabecular meshwork sample GTM304–04.

To access the data, click or select the words “Appendix 3.” This will initiate the download of a compressed (pdf) archive that contains the file.

## Appendix 4. Relative protein abundance: Trabecular meshwork sample GTM477–02.

To access the data, click or select the words “Appendix 4.” This will initiate the download of a compressed (pdf) archive that contains the file.

## Appendix 5. Average relative protein abundance over all DEX-treated TM cell Samples.

To access the data, click or select the words “Appendix 5.” This will initiate the download of a compressed (pdf) archive that contains the file.
